# Bacterial pathogens in *Ixodes ricinus* collected from lizards *Lacerta agilis* and *Zootoca vivipara* in urban areas of Wrocław, SW Poland– preliminary study

**DOI:** 10.1007/s10493-024-00927-2

**Published:** 2024-06-13

**Authors:** Dagmara Dyczko, Alicja Krysmann, Aleksandra Kolanek, Bartosz Borczyk, Dorota Kiewra

**Affiliations:** 1 Department of Microbial Ecology and Acaroentomology, Faculty of Biological Sciences, University of Wrocław, Przybyszewskiego 63/77, Wrocław, 51-148 Poland; 2 Faculty of Chemistry, Biotechnology and Food Science, Norwegian University of Life Sciences, NMBU, Ås, Norway; 3 Department of Geoinformatics and Cartography, Institute of Geography and Regional Development, Faculty of Earth Sciences and Environmental Management, University of Wrocław, pl. Uniwersytecki 1, Wrocław, 50-137 Poland; 4 Department of Evolutionary Biology and Conservation of Vertebrates, Faculty of Biological Sciences, University of Wrocław, Sienkiewicza 21, Wrocław, 50-335 Poland

**Keywords:** Bacterial pathogens, *Ixodes ricinus*, Lizards, Urban ecology, Wrocław

## Abstract

The aim of this study was to determine the level of infection of *Ixodes ricinus* ticks with pathogens (*Borrelia* spp., *Rickettsia* spp., and *Anaplasma* spp.) collected from *Lacerta agilis* and *Zootoca vivipara* lizards in the urban areas of Wrocław (SW Poland). The study was carried out in July-August 2020. Lizards were caught by a noose attached to a pole or by bare hands, identified by species, and examined for the presence of ticks. Each lizard was then released at the site of capture. Ticks were removed with tweezers, identified by species using keys, and molecular tests were performed for the presence of pathogens. From 28 lizards (17 specimens of *Z. vivipara* and 11 specimens of *L. agilis*) a total of 445 ticks, including 321 larvae and 124 nymphs, identified as *I. ricinus* were collected. A larger number of ticks were obtained from *L. agilis* compared to *Z. vivipara*. Molecular tests for the presence of pathogens were performed on 445 specimens of *I. ricinus*. The nested PCR method for the *fla* gene allowed the detection of *Borrelia* spp. in 9.4% of ticks, and it was higher in ticks from *L. agilis* (12.0%) than from *Z. vivipara* (1.0%). The RFLP method showed the presence of three species, including two belonging to the *B. burgdorferi* s.l. complex (*B. lusitaniae* and *B. afzelii*), and *B. miyamotoi*. The overall level of infection of *Rickettsia* spp. was 19.3%, including 27.2% in ticks collected from *Z. vivipara* and 17.0% from *L. agilis*. Sequencing of randomly selected samples confirmed the presence of *R. helvetica*. DNA of *Anaplasma* spp. was detected only in one pool of larvae collected from *L. agilis*, and sample sequencing confirmed the presence of *(A) phagocytophilum*. The research results indicate the important role of lizards as hosts of ticks and their role in maintaining pathogens in the environment including urban agglomeration as evidenced by the first recorded presence of *(B) miyamotoi* and *(A) phagocytophilum* in *I. ricinus* ticks collected from *L. agilis*. However, confirmation of the role of sand lizards in maintaining *(B) miyamotoi* and *A. phagocytophilum* requires more studies and sampling of lizard tissue.

## Introduction

Infections with tick-borne pathogens (TBP) are noted not only in wild areas but also with increasing frequency in urban in suburban areas across the world (Uspensky 2014; Król et al. 2016). The risk assessment of tick-borne diseases requires comprehensive studies involving ticks, hosts, and reservoir animals that are responsible for the maintenance of zoonotic pathogens. In Central Europe, in wild, suburban, and urban areas, *Ixodes ricinus* is the main vector of TBP, however, also other tick species like *Dermacentor reticulatus* and *I. hexagonus* can support the circulation of pathogens in the environment (Uspensky 2014). In urban areas, as hosts of adult ticks can serve both wild animals like deer, lagomorphs, foxes, and raccoons, which easily penetrate into green spaces inside cities as well as domesticated animals including dogs, and cats which are often infested by female ticks (Uspensky 2014; Król et al. 2015, 2016; Kocoń et al. 2023). The important role as hosts of immature stages play rodents and birds, especially ground-feeding songbirds, which also can be reservoir hosts for several tick-borne pathogens (Mihalca and Sándor 2013; Rizzoli et al. 2014; Eisen 2023). As hosts of immature ticks can serve also lizards, which often inhabit urban areas. The important role of lizards as *I. ricinus* hosts was documented for example in Italy, where the comparable prevalence of infestation by *I. ricinus* larvae on lizards *Podarcis muralis* and mice *Apodemus* spp. was documented, and even more, the infestation level by nymphs were significantly greater on lizards (Amore et al. 2007). However, the role of lizards in maintaining tick population and tick-borne pathogens (TBP) has been still underestimated compared to that of mammals and birds (Ekner et al. 2011; Rizzoli et al. 2014).

Among bacterial TBP most significant and well-researched are *Borrelia* spp., *Rickettsia* spp., and *Anaplasma* spp. (Rochlin and Toledo 2020; Hauck et al. 2020a). *Borrelia* spirochetes transmitted by hard ticks are responsible for Lyme borreliosis (LB) being the most frequently reported vector-borne disease in the Northern Hemisphere and for tick-borne relapsing fever (TBRF). LB spirochaetes grouped into *Borrelia burgdorferi* sensu lato (s.l.) complex comprises over 20 named and proposed genospecies (Wolcott et al. 2021). Several of them are known to cause borreliosis in humans and animals (Sykes and Makiello 2017; Wolcott et al. 2021). In the relapsing fever group, an increasingly noted species is *Borrelia miyamotoi*, detected first in 1995 in ticks from Japan (Fraenkel et al. 2002). Disease caused by *B. miyamotoi* is similar to RF but can also bear similarities to borreliosis (like skin lesions) (Platonov et al. 2011). *B. miyamotoi* is also transmitted by *Ixodes* ticks but unlike.

*B. burgdorferi* s.l. it can be transmitted not only in a transstadial way but also via transovarial transmission (Rollend et al. 2013). *Rickettsia* species are grouped into four main groups: spotted fever group (SFG), typhus group (TG), transitional group (TRG), and ancestral group (AG) (Kim 2022). The SFG of *Rickettsia* frequently infects ticks and causes tick-transmitted rickettsioses (Portillo et al. 2015). In Central Europe, SFG of *Rickettsia* which are potentially able to affect humans include among other *Rickettsia*, which are transmitted by *I. ricinus* like *R. helvetica*, and *R. monacensi*s (Karbowiak et al. 2016a). *Anaplasma* spp., similar to *Rickettsia*, are intracellular obligate pathogens that can be transmitted by vector (Bakken et al. 2008). From a medical point of view, the most important is *Anaplasma phagocytophilum*, an agent of human granulocytic anaplasmosis (HGA) (Dumler et al. 2005). The infection with *A. phagocytophilum* is also detected in several mammalian species, including domesticated animals like ruminants, horses, dogs, and cats (Karbowiak et al. 2016b). The current study was undertaken to evaluate the prevalence of *Borrelia* spp., *A. phagocytophilum*, and *Rickettsia* spp. in ticks collected from lizards in urban areas of Wrocław, SW Poland.

## Materials and methods

### Ticks collection

**Fig. 1:**
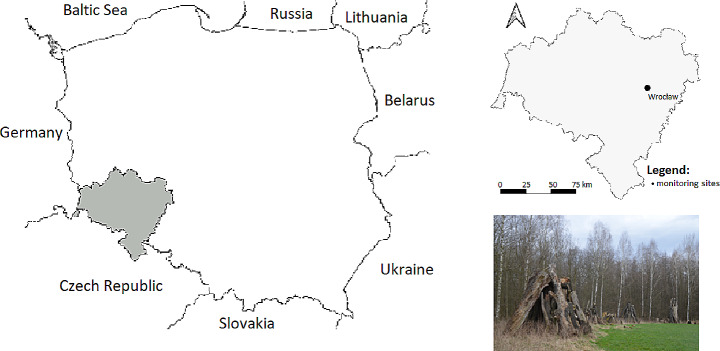
Lizards catching place located in Rędziński Forest, Wrocław (SW Poland)

The study was carried out in July-August 2020 in a mid-forest meadow in the urban forest (Rędziński Forest) in the northern part of Wrocław, SW Poland (Fig. 1). The Rędziński Forest is located alongside two rivers: Oder and Widawa, and it is second in size forest within the city borders, covering an area of 400 ha. Part of the area belongs to a protected area designated as a Natura 2000 site under the name of Widawa Valley (Filipiak and Zaręba 2014). This study area is an important part of city green areas popular for recreation (Filipiak and Zaręba 2014; Turzanska and Turowicz 2014). The Rędziński Forest is inhabited by valuable species of wild animals, and it is also frequently visited by dogs walked by their owners. Two species of co-existing lizards: the sand lizard *Lacerta agilis* and the common or viviparous lizard *Zootoca vivipara* were caught by a noose pole or by bare hands (Bennett et al. 2001). Captured lizards were examined for the presence of ticks, which were removed with forceps and stored in 70% ethanol. After tick collection lizards were released back into the habitat. Collected ticks were identified to the species level by the use of the key to Ixodida identification (Estrada-Peña et al. 2017). The study was carried out under a permit from the Polish Authorities No. WPN.6401.212.2019.MH.1.

### Molecular methods

All collected nymphs of *I. ricinus* were tested separately. In the case of *I. ricinus* larvae, a total of 107 pools containing three specimens, were prepared, including 23 pools of larvae collected from *Z. vivipara*, and 84 pools of larvae obtained from *L. agilis*. DNA extraction from ticks was done with the ammonium method by Guy and Stanek (1991). Homogenized ticks (nymphs separately, and larvae pooled by 3 specimens) were placed in 100 µl of 0,65 M of ammonium hydroxide (NH_4_OH) and heated for 20 min in 100℃ in closed tubes, followed by 10 min in 100 °C in open tubes. After the extraction tubes were centrifuged at 11 000 rpm for 3 min, the supernatant was stored at -20℃ until molecular methods. For detection of *Borrelia* spp. *Anaplasma* spp, and *Rickettsia* spp. the specific nested PCR were used (Table 1). *Borrelia* spp. detection targeted *flaB* gene encoding flagellin (Wodecka et al. 2009), *Anaplasma* spp. detection targeted 16 S rRNA (Massung et al. 1998), and *Rickettsia* spp. detection targeted *glt*A gene, encoding citrate synthase (Prakash et al. 2012; Mitková et al. 2015). All nested PCR were carried out with the use of PCRMix Plus (A&A Biotechnology) and Thermal Cycler T100 (BioRad T100™ Thermal Cycler), reactions were followed by electrophoresis in 1,5% agarose gel (30 min, 100 V), and results were observed under UV light. Randomly selected nested PCR positive samples, including 3 samples of *Rickettsia*, 4 samples of *Borrelia* and the only Anaplasma detected, were prepared with the use of EPPiC kit (A&A Biotechnology) and sent for sequencing to MACROGEN (Netherlands). Obtained sequences were compared to existing ones in GenBank with the use of BLAST.

**Table 1:** Nested PCR used for pathogen detection in *Ixodes ricinus* ticks collected from lizards

Pathogen	Gen detected	Primers	Product size	Literature
*Borrelia* spp	*flaB*	132f: 5’-TGGTATGGGAGTTTCTGG-3’	605 bp	Wodecka et al. 2009
		905r: 5’-TCTGTCATTGTAGCATCTTT-3’,		
		220f: 5’-CAGACAACAGAGGGAAAT-3’		
		824r: 5’- AAGTCTATTTTGGAAAGCACC-3’		
*Anaplasma* spp	16 S rRNA	ge3a: 5’-CACATGCAAGTCGAACGGATTATTC-3’,	546 bp	Massung et al. (1998)
		ge10r: 5’-TTCCGTTAAGAAGGATCTAATCTCC-3’		
		ge9f: 5’-AACGGATTATTCTTTATAGCTTGCT-3’,		
		ge2: 5’-GGCAGTATTAAAAGCAGCTCCAGG-3’		
*Rickettsia* spp	gltA	877p: 5´- GGGGACCTGCTCACGGCGG-3´	338 bp	(Prakash et al. 2012; Mitková et al. 2015).
		1258n: 5´- ATTGCAAAAAGTACAGTGAACC-3’		
		896p: 5’-GGCTAATGAAGCAGTGATAA-3’		
		1233n: 5’-GCGACGGTATACCCATAGC-3’		

For differentiation genospecies of *Borrelia* spp. nested PCR positive samples were analysed by RFLP with the use of restriction enzyme HpyF3I (FastDigest HpyF3I, ThermoScientific), which recognises CTNAG sequence (Wodecka 2011). Restriction patterns obtained after electrophoresis in 3% agarose gel (75 min, 65 V) were observed under UV light and assigned to appropriate genospecies.

### Statistical analysis

To assess the significance of obtained results χ2 test with *p* < 0,05 and 95% confidence intervals (95% CI) was used. To assess the discrepancy in the number of ticks parasitising certain lizard species, the standard deviation (σ) was calculated, based on the average number of parasitising ticks. To assess the level of infection in larvae ticks, Minimal Infection Rate (MIR) was calculated, assuming that one positive larvae gives a positive pool, and in the positive pool, there was only one positive larvae.

$$ MIR=\left(\left(number of positive larvae pools\right):\left(total number of tested larvae\right)\right)*100\%$$

## Results

From 28 lizards (17 specimens of *Zootoca. vivipara* and 11 specimens of *Lacerta agilis*) a total of 445 ticks, including 321 larvae and 124 nymphs(Table 2). All ticks collected from these lizards were identified as *Ixodes ricinus*. A larger number of ticks were obtained from *L. agilis* (342 ticks, including 252 larvae, and 90 nymphs) compared to *Z. vivipara* (103 ticks, including 69 larvae and 34 nymphs). The average number of ticks infested *L. agilis* was 31.1, and *Z. vivipara* 6.1 with the highest number of 90 ticks on a single *L. agilis* and 19 ticks on *Z. vivipara.*

**Table 2:** Level of lizard infestation with *Ixodes ricinus* ticks (σ**-**standard deviation)

Species	Number of lizards	Total number of ticks	Larvae	Nymphs	Average number of ticks (σ)	Highest number of ticks
*Zootoca vivipara*	17	103	69	34	6.1 (4,5)	19
*Lacerta agilis*	11	342	252	90	31.1 (23,2)	90

Total infection rate of *Borrelia* spp. for all 445 *I. ricinus* ticks, calculated for larvae and nymphs together (assuming that one positive larvae gives a positive pool, and in the positive pool there was only one positive larvae) was 9.4% (42/445, 95% CI: 6.7–12.2) (Table 3), and it was higher in ticks from *L. agilis* (12.0%, 41/342, 95% CI: 8.6–15.4) than from *Z. vivipara* (1.0%, 1/103, 95% CI: 0-2.9). MIR of *Ixodes ricinus* larvae collected from both species of lizards was 4.0%, however, spirochaetes were only present in larvae from *L. agilis* (MIR = 5.2%). The total infection rate of nymphs (collected from both species of lizards) with *Borrelia* spp. was 23.4% (29/124, 95% CI: 15.9–30.8). There was a statistically higher infection rate in nymphs from *L. agilis* (31.1%, 28/90, 95% CI: 21.6–40.7) than in nymphs from *Z. vivipara* (2.9%, 1/34, 95% CI: 0-8.6) (χ2 = 10,93, df = 1, *p* < 0,001). RFLP analysing of 42 positive products of nested PCR allows the identification of 3 restriction patterns, including two from *B. burgdorferi* s.l. complex (*B. lusitaniae* and *B. afzelii*), and *B. miyamotoi* (Table 4). Sequencing four randomly selected samples identified using RFLP as *B. lusitaniae* confirmed their species identification. The dominant species of *Borrelia* was *B. lusitaniae* identified in 88.0% samples, including 69.0% mono-infections and 19.0% coinfection with *B. miyamotoi*. *B. lusitaniae* was the only *Borrelia* species detected in nymphs (38.0%) of both lizard species. *B. afzelii* was detected only in one nymph (2.4%) collected from *L. agilis. B. miyamotoi* was detected only in nymphs collected from *L. agilis* both as single specimens of *Borrelia* (9.5%) and coinfection with *B. lusitaniae* (19.0%).

**Table 3:** Pathogens detected in *Ixodes ricinus* ticks from lizards *Lacerta agilis* and *Zootoca vivipara*

Species of lizard	Developmental stage of ticks	No. of tested ticks	No. of infected/ % (95% CI)		
			Borrelia spp.	Anaplasma spp.	Rickettsia spp.
*Lacerta agilis*	larvae	252	13/5.2 (2.4–7.9)	1/0.4 (0-1.2)	18/7.1 (3.9–10.3)
	nymphs	90	28/31.1 (21.6–40.7)	0/0.0	40/44.4 (34.2–54.7)
**Total**		**342**	**41/12.0** **(8.6–15.4)**	**1/0.3** **(0-0.9)**	**58/17.0** **(13.0-20.9)**
*Zootoca vivipara*	larvae	69	0/0.0	0/0.0	16/23.2 (13.2–33.1)
	nymphs	34	1/2.9 (0-8.6)	0/0.0	12/35.3 (19.2–51.4)
**Total**		**103**	**1/1.0** **(0-2.9)**	**0/0.0**	**28/27.2** **(18.6–35.8)**

**Table 4:** *Borrelia* species identified in *I. ricinus* ticks collected from lizards

Lizard species	Tick stage	B. lusitaniae	B. miyamotoi	B. afzelii	B. lusitaniae/ B. miyamotoi
***Z. vivipara***	nymphs	1	-	-	-
***L. agilis***	larvae	13	-	-	-
	nymphs	15	4	1	8
**Total**		29 (69.0%)	4 (9.5%)	1 (2.4%)	8 (19.0%)

The total infection rate of *Rickettsia* spp. was 19.3% (86/445, 95% CI: 15.7–23.0), including 27.2% (28/103, 95% CI: 18.6–35.8) in ticks collected from *Z. vivipara* and 17.0% (58/342, 95% CI: 13.0-20.9) from *L. agilis*. MIR for *I. ricinus* larvae collected from both lizard species was 10,5% and was higher for *Z. vivipara* (23,2%) than for *L. agilis* (7,1%) (χ2 = 13.557, df = 1, *p* = 0.0002). The prevalence for nymphs was 42.0% (52/124, 95% CI: 33.3–50.6), including 35.3% (12/34, 95% CI: 19.2–51.4) for nymphs collected from *Z. vivipara* and 44.4% (40/90, 95% CI: 34.2–54.7) from *L. agilis*, however the difference in the prevalence of *Rickettsia* spp. detected in nymphs was insignificant (χ2 = 0.849, df = 1, *p* = 0.849). Sequencing of three randomly selected samples confirmed the presence of *R. helvetica*.

DNA of *Anaplasma* spp. was detected only in one pool of larvae collected from *L. agilis.* Sample sequencing confirmed the presence of *A. phagocytophilum*.

*Borrelia* and *Rickettsia* coinfection was found in 13 cases (2 pools of larvae and 11 nymphs) of ticks collected only from *L. agilis*. Among *Borrelia* identified in coinfection were *B. lusitaniae* (2 pools of larvae and 9 nymphs), *B. lusitaniae* and *B. miyamotoi* (one nymph), and *B. afzelii* (one nymph),

## Discussion

In Poland, *Lacerta agilis* and *Zootoca vivipara* are among the most numerous and frequently occurring reptile species (Ekner et al. 2008; Borczyk et al. 2022; Jurczyk and Borczyk 2022). *Lacerta agilis* can be found in a wide range of habitats and occur in western, central, and eastern Europe as well as in western and central Asia, while the range of *Z. vivipara* covers most of Europe and northern Asia. However, especially in Western Europe, both species are endangered and declining (Ekner et al. 2008). Our study confirms the occurrence of *L. agilis* and *Z. vivipara* in urban areas of Wrocław (SW Poland) and indicates their important role as hosts of immature stages of *Ixodes ricinus*. The role of *L. agilis* and *Z. vivipara* in maintaining *I.ricinus* population in urban areas was also documented in western Poland (Gwiazdowicz et al. 2020). In our study, we find that the importance of *L. agilis* in maintaining the tick population seems to be much higher compared to *Z. vivipara*. The greater number of ticks infesting *L. agilis* compared to *Z. vivipara* (in our study we collected an average of 31.1 ticks per one infested specimen of *L. agilis* and 6.1 ticks per *Z. vivipara*), may be related to greater mobility of *L. agilis* than *Z. vivipara* (Ekner et al. 2008) as well as in the differences in microhabitat preferences between the species (Borczyk et al. 2022). In contrast, Gwiazdowicz et al. (2020) found that females of *L. agilis* had lower infestation than *Z. vivipara*. The sex of the lizards may have a significant impact on the level of infestation because Dudek et al. (2016) found that males of *L. agilis* lizards are more frequently parasitized by ticks than females. Wieczorek et al. (2020), on the other hand, analyzing ticks attacking *L. agilis* on the outskirts of the city (Zielona Góra, Poland) did not confirm the correlation between the sex of lizards and the level of infestation by ticks, but they draw attention to body mass. The significant role of *L. agilis* in maintaining *I. ricinus* population in Poland was reported previously (Gryczyńska-Siemiątkowska et al. 2007; Gwiazdowicz et al. 2020; Ekner et al. 2011). *L. agilis* as a host of *I. ricinus* was also found in other European countries, including Hungary (Földvári et al. 2009), the Netherlands (Tijsse-Klasen et al. 2010), Germany (Richter and Matuschka 2006), Sweden (Olsson et al. 2000).

In our study, DNA of *Borrelia* spp. was detected in *I. ricinus* collected from both lizard species, however, a higher percentage of infected ticks was found in *L. agilis* than *Z. vivipara*, and from *L. agilis* both larvae and nymphs were infected, while from *Z. vivipara* only one nymph. Among identified *Borrelia* the dominant genospecies was *Borrelia lusitaniae* (88.0% positive samples). *Borrelia lusitaniae* was also the only identified *Borrelia* species in nymph from *Z. vivipara* and in larvae from *L. agilis*. A greater diversity of *Borrelia* was found in nymphs from *L. agilis*, because in addition to *B. lusitaniae*, also *B. afzelii* and *B. miyamotoi* were detected. Moreover, *B. miyamotoi* occurred in 28.5% of cases with *Borrelia*, including 9.5% single *Borrelia* infection and 19.0% co-infection with *B. lusitaniae*. According to our knowledge, this is the first report of the presence of *B. miyamotoi* in ticks collected from *L. agilis.* In an earlier study conducted in the area of Ranzenberg Mountain, Germany by Richter and Matuschka (2006), the occurrence of *B. miyamotoi* was observed in one nymph obtained from a common wall lizard (*Podarcis muralis*). However, the presence of *B. miyamotoi* in nymphs collected from lizards may be the result of acquiring the pathogen from the blood of the first host on which the tick was parasitized in the larva stage. It cannot also be excluded, that *B. miyamotoi* was transovarially to larvae and from larvae to nymphs transstadially transmitted, because *B. miyamotoi*, unlike *B. burgdorferi*, is capable to transovarial transmission in ticks of the *Ixodes* species (Rollend et al. 2013). Confirmation of the role of sand lizards in maintaining *B. miyamotoi* acquired more studies and sampling of lizard tissue.

The presence of *B. lusitaniae* in *I. ricinus* collected from *L. agilis* is detected also in other studies. Ekner et al. (2011) during a study carried out in the years 2008–2009 in the Barycz Valley, in Polan found DNA of *B. burgdorferi* s.l. in 4.1% ticks, including predominant *B. lusitaniae* and less frequently *B. burgdorferi* s.s. Gryczyńska-Siemiątkowska et al. (2007) in 2002–2003 in the Mazurian Lake region (Northeastern Poland) detected in 4.7% ticks parasitized sand lizards DNA of *B. burgdorferi* s.l., including *B. afzelii*, *B. garinii* and *B. burgdorferi* s.s. In the area near the town Odolanów, SW Poland, prevalence reached 6% with *B. lusitaniae* as the dominant genospecies and less frequently *B. valaisiana* (Majláthová et al. 2008). In Slovakia, the only detected genospecies in nymphs were *B. lusitaniae*, and *B. lusitaniae* and *B. afzelii* in larvae (Majláthová et al. 2008). While in the Netherlands, *B. burgdorferi* s.s and *Borrelia afzelii* were identified in ticks collected from sand lizards and no *B. lusitaniae* was detected (Tijsse-Klasen et al. 2010).

Xenodiagnosis, being the strongest method used for estimating vector competence, confirms the reservoir competence of *Psammodromus algirus* for *B. lusitaniae* (Dsouli et al. 2006; Wolcott et al. 2021). However, reservoir competence for *B. lusitaniae* seems to have more lizard species, including *L. agilis*, and the role of *L. agilis* in maintaining *B. lusitaniae* is evidenced by lizard studies. In Poland, DNA of *B. lusitaniae* was detected in 1.2% of *L. agilis* collected in the Barycz Valley (Ekner et al. 2011). In Hungary, *B. lusitaniae* was found in tissue samples of green lizards *Lacerta viridis*, Balkan wall lizards *Podarcis taurica*, and *L. agilis*, although the presence of *Borrelia* in ticks collected from *L. agilis* has not been confirmed (Földvári et al. 2009). Biopsy specimens from *L. agilis* positive for *B. lusitaniae* were also found in 45% of the sand lizards from Slovakia and 57% from Romania, although the same studies have not confirmed the presence of *B. burgdorferi* s.l. in samples of *L. agilis* from Poland (Majláthová et al. 2008). Many studies confirmed, however, the presence of *B. lusitaniae* both in lizard and parasiting ticks. *Borrelia* both in *L. agilis* and *I. ricinus* was found in Slovakia and in Romania (Majláthová et al. 2008), in the Czech Republic in *Lacerta viridis* and *I.ricinus* (Musilová et al. 2022), in Italy, in *Podarcis muralis* and *I. ricinus* (Amore et al. 2007), in Slovakia in *Lacerta viridis* and *I. ricinus* (Majláthová et al. 2006), in Italy in *Podarcis siculus* and *I. ricinus* (Mendoza-Roldan et al. 2019).

The most common among the bacterial pathogens detected in our study was *Rickettsia* spp. because 19.3% of ticks (both larvae and nymphs) were infected, including 27.2% ticks from *Z. vivipara* (23.2% larvae, 35.3% nymphs) and 17.0% from *L. agilis* (7.1% larvae, 44.4% nymphs). *Rickettsia* spp. found in ticks collected from sand lizards in the Netherlands were also identified as *R. helvetica* (Tijsse-Klasen et al. 2010) and in Slovakia in *L. viridis* (Václav et al. 2011). A high level of *Rickettsia* infection in ticks from lizards was also found in the Iberian Peninsula, where Kubelová et al. (2015) detected rickettsial DNA in 47% of nymphs and 31.6% of larvae collected from Iberian lizard *Lacerta schreiberi*. However, taking into account that most *Rickettsia* species can be transmitted transovarially, the infection in larvae may occur without the need to parasitize on the reservoir hosts.

Unexpectedly, we detected DNA of *Anaplasma phagocytophilum* in *I. ricinus* larvae collected from *Lacerta agilis.* However recently, the possibility of transovarial transmission of *A. phagocytophilum* is being considered because *A. phagocytophilum* was found in questing *I. ricinus* larvae (Jahfari et al. 2014, Hauck et al. 2020b). It can suggest that transovarial transmission in ticks might occur, however with low efficiency. The possibility of the occurrence of *Anaplasma* in *L. agilis* is also supported by molecular studies conducted by Ekner et al. 2011), who found DNA of Anaplasmataceae both in *I. ricinus* collected from *L. agilis*, and additional, for the first time, in *L. agilis.* The occurrence of *Anaplasma*/*Ehrlichia* spp. was also found in ticks from sand lizards in the Netherlands. however, the prevalence was significantly lower than in questing ticks (Tijsse-Klasen et al. 2010). Whereas, a relatively high percentage of infection with *A. phagocytophilum* of ticks collected from *L. viridis* found Václav et al. (2011) in Slovakia, who estimated the infection rate at 13.1% for nymphs and 8.7% for larvae.

Knowledge of transmission ways is crucial for understanding the complete lifecycle of *A. phagocytophilum*, including the possible role of lizard in *A. phagocytophilum* maintenance.

## Conclusion

*Lacerta agilis* and *Zootoca vivipara* in peri-urban areas are among the important host of immature stages of *Ixodes ricinus*. However, the role of *L. agilis* seems to be greater compared to *Z. vivipara*. The high levels of *B. lusitaniae* infections in ticks that had fed on *L. agilis* lizards may point to implicates this species of lizard in the transmission cycle of *B. lusitaniae*. However, the presence of *B. miyamotoi* and *A. phagocytophilum* detected for the first time in ticks collected from this lizard species requires further research to confirm their participation in the transmission cycle of these pathogens.
